# Severe Acute Respiratory Syndrome Coronavirus 2 Infection in Pregnancy. A Non-systematic Review of Clinical Presentation, Potential Effects of Physiological Adaptations in Pregnancy, and Placental Vascular Alterations

**DOI:** 10.3389/fphys.2022.785274

**Published:** 2022-03-30

**Authors:** Paola Ayala-Ramírez, Marcelo González, Carlos Escudero, Laura Quintero-Arciniegas, Fernanda R. Giachini, Raiany Alves de Freitas, Alicia E. Damiano, Reggie García-Robles

**Affiliations:** ^1^School of Medicine, Human Genetics Institute, Pontificia Universidad Javeriana, Bogotá, Colombia; ^2^Group of Research and Innovation in Vascular Health (GRIVAS Health), Chillan, Chile; ^3^Laboratorio de Investigación Materno-Fetal (LIMaF), Departamento de Obstetricia y Ginecología, Facultad de Medicina, Universidad de Concepción, Concepción, Chile; ^4^Laboratory of Vascular Physiology, Department of Basic Sciences, Faculty of Sciences, Universidad del Bio-Bio, Chillan, Chile; ^5^Perinatal Medicine Seedbed, School of Medicine, Pontificia Universidad Javeriana, Bogotá, Colombia; ^6^Department of Physiological Sciences, School of Medicine, Pontificia Universidad Javeriana, Bogotá, Colombia; ^7^Institute of Biological Sciences and Health, Federal University of Mato Grosso, Barra do Garças, Brazil; ^8^Institute of Biological Sciences, Federal University of Goias, Goiânia, Brazil; ^9^Laboratorio de Biología de la Reproducción, Instituto de Fisiología y Biofísica Bernardo Houssay (IFIBIO)- CONICET- Facultad de Medicina, Universidad de Buenos Aires, Buenos Aires, Argentina; ^10^Cátedra de Biología Celular y Molecular, Departamento de Ciencias Biológicas, Facultad de Farmacia y Bioquímica, Universidad de Buenos Aires, Buenos Aires, Argentina

**Keywords:** COVID-19, pregnancy, renin–angiotensin–aldosterone system, coronavirus in pregnancy, placenta

## Abstract

In December 2019, the novel Severe Acute Respiratory Syndrome Coronavirus 2 (SARS-CoV-2) rapidly spread to become a pandemic. To date, increasing evidence has described the potential negative impact of SARS-CoV-2 infection on pregnant women. Although the pathophysiology of coronavirus disease 2019 (COVID-19) is not entirely understood, there is emerging evidence that it causes a severe systemic inflammatory response associated with vascular alterations that could be of special interest considering some physiological changes in pregnancy. Additionally, these alterations may affect the physiology of the placenta and are associated with pregnancy complications and abnormal histologic findings. On the other hand, data about the vaccine against SARS-CoV-2 are limited, but the risks of administering COVID-19 vaccines during pregnancy appear to be minimal. This review summarizes the current literature on SARSCoV2 virus infection, the development of COVID-19 and its relationship with physiological changes, and angiotensin-converting enzyme 2 (ACE2) function during pregnancy. We have particularly emphasized evidence coming from Latin American countries.

## Introduction

In December 2019, an unknown etiology outbreak of pneumonia was described in Wuhan, China. By January 2020, a new type of coronavirus was identified as the primary cause of these pneumonia cases. Severe Acute Respiratory Syndrome Coronavirus 2 (SARS-CoV-2) was identified as a beta coronavirus, the same subtype as its predecessors SARS-CoV and the Middle East Respiratory Syndrome (MERS-CoV). With the rapid spread of cases, the WHO declared a pandemic of coronavirus disease of 2019 (COVID-19) on March 11, 2020.

Severe Acute Respiratory Syndrome Coronavirus 2 causes essential alterations in the cardiovascular system beyond the initial damage to the respiratory system (Zheng et al., 2019). Alterations include the vascular endothelium-mediated recruitment of inflammatory leukocytes that contribute to tissue damage and cytokine release. These alterations are critical drivers of the acute respiratory distress syndrome and disseminated intravascular coagulation and COVID-19-associated cardiovascular complications.

During pregnancy, physiological adaptive changes in the immune, respiratory, cardiovascular, and coagulation systems could modulate COVID-19 presentation. As pregnant women are at high risk of complications and severe disease from infection with other coronaviruses, they were identified as a vulnerable group and were advised to take additional precautions as the COVID-19 pandemic unfolded. Accordingly, later evidence has shown that pregnant women seem to be associated with a greater susceptibility to contagion (Wastnedge et al., 2021), presenting more severe forms of the disease (Liu H. et al., 2020), or a high risk for pregnancy complications (di Mascio et al., 2020). In addition, data have confirmed SARS-CoV-2 vertical transmission, although short- and long-term sequels are still under investigation. Despite that, histological studies of placentas from SARS-CoV-2 positive pregnant women showed poor uterine-placental perfusion, with signs of placental infarcts, atheromas in the decidua vessels chorioangioma, and edema in placental villi (Mulvey et al., 2020; Shanes et al., 2020).

This review summarizes the current literature on SARS-CoV-2 virus infection in pregnancy. In addition, we describe pregnancy-associated adaptations in the angiotensin-converting enzyme 2 (ACE2), a protein identified as SARS-CoV-2 receptor, which could influence the presentation of COVID-19. This review particularly emphasized evidence coming from Latin American countries.

## Pathophysiology of Severe Acute Respiratory Syndrome Coronavirus 2 Infection

Severe Acute Respiratory Syndrome Coronavirus 2 is an encapsulated positive single-stranded RNA virus, a coronavirus (Elias et al., 2021). The coronavirus family comprises numerous viruses with the capacity to infect several species (Channappanavar et al., 2014). So far, some of them, including coronaviruses 229E and NL63, display the capacity to affect humans, generating symptoms of a standard cold (Rabi et al., 2020). The comprising ability of these viruses to bind host cells requires a projection of the membrane structure, called “*spike*.” This transmembrane structure is composed of a trimetric glycoprotein protuberance, composed of two subunits. The first one, S1, is the binding region to the host cell receptor and, S2 is the region where the virus and the host cellular membranes may fuse (Tang et al., 2020). Person-to-person transmission has been demonstrated through drops, contact, and aerosols spread, but less frequently due to fecal-oral and fomites transmission. The incubation period is around 5 days (5.79–6.97 days) (Elias et al., 2021). For diagnosis, the standard gold technique is the real-time PCR (RT-PCR), which detects the presence of SARS-CoV-2 virus RNA.

At the onset of COVID-19, the most common symptoms are fever, cough, and fatigue, while other symptoms include sputum production, headache, diarrhea, dyspnea, and lymphopenia (Rothan and Byrareddy, 2020). Once inside the host, SARS-CoV-2 activates the innate and adaptive immune responses and elicits a pronounced lymphopenia due to impaired lymphopoiesis and increased lymphocyte apoptosis (Wiersinga et al., 2020). Around 7–8 days after the onset of signs/symptoms, some individuals progress to a more clinically compromised condition and develop pneumonia with respiratory distress requiring hospitalization. In some cases, sepsis appears around days 9–10, and severe acute respiratory syndrome occurs on days 9–12. In some individuals, the start of mechanical ventilation and admission to the Intensive Care Unit (ICU) is required approximately at 10.5 days. Complications, such as heart or kidney injury, usually occur on day 15, secondary infection on day 17, and death or recovery on days 19–22 (Huang et al., 2020; Zhou F. et al., 2020). Some factors that seem to be associated with a higher risk of infection and severe disease are age (>70 years old), male sex, tobacco use, and presence of comorbidities, such as chronic diseases (Cai, 2020; Jin et al., 2020; Jordan et al., 2020; Zheng et al., 2020). Additionally, a high score on the Sequential Organ Failure Assessment (SOFA) and values >1 μg/ml for D-dimer have been associated with higher mortality (Zhou F. et al., 2020).

**FIGURE 1:**
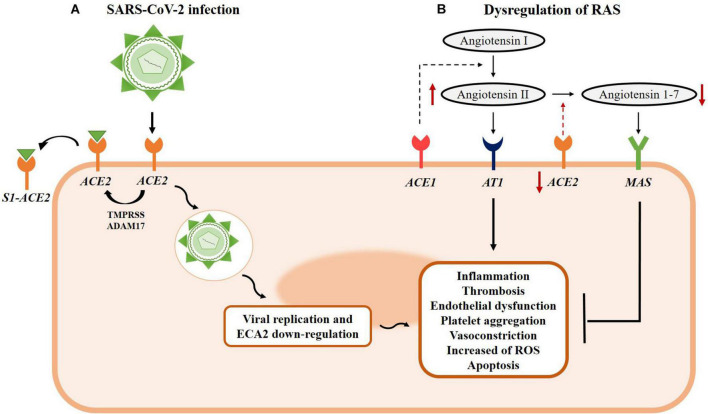
Mechanism of Severe Acute Respiratory Syndrome Coronavirus 2 (SARS-CoV-2) infection and RAS system dysregulation. **(A)** SARS-CoV-2 uses the ACE2 receptor to enter human cells. Spike proteins can be cleaved by different host proteases to bind to ACE2 receptors. The S1-ACE2 interaction triggers the cleavage of ACE2 through transmembrane protease, serine 2 (TMPRSS) proteases or by ADAM17/TACE, resulting in the release of S1-ACE2 interaction from the host cells. The S1 angiotensin-converting enzyme 2 (ACE2) complex is followed by membrane fusion and subsequent viral endocytosis, which releases the viral genome into the cytoplasm, ACE2 downregulation, and pathological cellular effects. **(B)** The SARS-CoV-2 infection process contributes to RAS dysregulation due to ACE2 downregulation and, consequently, reduced conversion of Ang 1–7 from angiotensin II (Ang II) and increased levels of Ang II. The balance between ACE1 and ACE2 is necessary to maintain physiological conditions and the production levels of Ang II and Ang 1–7, respectively. The Ang II/AT1 interaction results in long-term exacerbated vasoconstriction, inflammation, thrombosis, and epithelial dysfunction. On the other hand, the Ang 1–7/MAS complex counteracts the harmful effects of Ang II by inducing vasodilation, anti-inflammatory effects, and tissue repair. In SARS-CoV-2 infection, the upregulation of Ang II leads to several long-term detrimental effects, while the lack of Ang 1–7 reduces the protective and counter-regulatory activities of the effects triggered by Ang II.

The mechanisms of SARS-CoV-2 invasion of the host system are illustrated in Figure 1. Briefly, the coronavirus virion has structural proteins: nucleocapsid (N), membrane (M), envelope (E), and spike (S) proteins. The entry steps of the viral particles—encompassing attachment to the host cell membrane and fusion—are mediated by the S glycoprotein. S protein is assembled as a homotrimer and is inserted in multiple copies into the virion membrane giving it its crown-like appearance. In addition, the furin-like proteases, transmembrane protease, serine 2 (TMPRSS2), and cathepsin L are involved in the virus invasion process. First, the S protein binds to the ACE2 receptor, after which the virus penetrates the host cells by endocytosis (S1 region) or membrane fusion (S2 region). Cleavage of the S1–S2 boundary is necessary for initiating the membrane-fusion process. After S1–S2 is cleaved, the S2 site activates the fusion process either by TMPRSS2 on the cell surface or by cathepsins in endosomes. Then, the fusion between viral and cellular membranes forms a pore through which viral RNA is released into the host cell cytoplasm for uncoating and replication (Zhou P. et al., 2020; Jackson et al., 2021).

Interestingly, the main characteristic of COVID-19 is endothelial cell damage (Evans et al., 2020). Thus, infection with SARS-CoV-2 reduces ACE2-mediated regulation of vascular tone and causes endothelial dysfunction at multiple levels, such as inflammatory activation, cytokine storm, leukocyte infiltration, increased permeability, thrombosis, platelet aggregation, vasoconstriction, reactive oxygen species (ROS) production, and apoptosis (Evans et al., 2020).

## Angiotensin-Converting Enzyme 2 and the Implication of the Renin-Angiotensin System on Coronavirus Disease 2019

Angiotensin-converting enzyme 2 is constitutively expressed in several tissues, such as the lung, heart, kidney, and blood vessels (Zou X. et al., 2020). Therefore, SARS-CoV-2 has a particular tropism for the pulmonary system, initiating pronounced respiratory symptoms (Jia et al., 2005). In addition, the renin-angiotensin system is a critical modulator of vascular function, as a key target of the SARS-CoV-2 infection (Watanabe et al., 2005; Fraga-Silva et al., 2013; Santos, 2014; Sanchis-Gomar et al., 2020). In particular, ACE2 is a pleiotropic peptidase that metabolizes Angiotensin II (Ang II) to Ang 1–7, two peptides with divergent physiological functions. Thus, while Ang II evokes vasoconstrictive, proliferative, and angiogenic effects, Ang 1–7 elicits anti-proliferative, anti-angiogenic, and vasodilator functions (Bharadwaj et al., 2011). Moreover, ACE2 has a protective effect on the endothelium and improves endothelial function, apparently mediated by Ang 1–7 production (Lovren et al., 2008; Fraga-Silva et al., 2013; Figure 1). The relevance of ACE2 has been confirmed in animals deficient in this enzyme. Those animals exhibit increased oxidative stress and pro-inflammatory cytokines, compromising cardiovascular function (Wang et al., 2020). Likewise, previous studies on the coronavirus-induced severe acute respiratory syndrome, sepsis, or acid aspiration-induced lung injury have shown that dysfunction of the renin-angiotensin system is involved in these conditions (Zhang et al., 2018; Li H. et al., 2020; Rezaei et al., 2021). Huang et al. (2014) reported that high plasma levels of Ang II were associated with the severity of the disease and also those high levels predicted fatal outcomes due to the H7N9 influenza virus. In Kuba et al. (2005) described that the function of ACE2 is impaired by the binding of viral protein S, enhancing the Ang II circulating levels, and leading to hemodynamic alterations characterized by vasoconstriction.

The critical role of ACE2 in the COVID-19 pathophysiology raised speculations about ACE2 as a therapeutic target and the implications of the use of renin-angiotensin system inhibitors. Since ACE2 inhibitors and Ang II AT1 receptor antagonists have the potential to increase ACE2 expression, which may aggravate COVID-19, we recommend critical publications regarding the use of renin-angiotensin inhibitors and COVID-19 (Bavishi et al., 2020; Peiró and Moncada, 2020; Sanchis-Gomar et al., 2020; Sommerstein et al., 2020; Vaduganathaan et al., 2020). Yet the presence of the soluble and active isoform of ACE2 may be beneficial in COVID-19 patients since soluble ACE2 may compete with the membrane enzyme, limiting its role as a SARS-CoV-2 receptor, and decreasing the Ang II plasma levels (Khodarahmi et al., 2021). As a therapeutic target, the restoration of ACE2 through the administration of recombinant ACE2 may reverse the lung-injury process (Monteil et al., 2020, 2021; Siriwattananon et al., 2021; Zhang et al., 2021).

Therefore, several studies emphasize the critical role of ACE2 in viral infection, the clinical presentation of COVID-19, the potential use of its products (i.e., metabolites) as biomarkers. In addition, the use of drugs that modulate ACE2 has to be taken into account in the treatment and clinical evolution of COVID-19.

## Pregnancy Is a Physiological Modulator of Angiotensin-Converting Enzyme 2 Expression and Activity

Two physiological conditions can modulate ACE2 levels and activity: aging and pregnancy (AlGhatrif, 2020; Huang et al., 2020; Ludvigsson, 2020; Zhou F. et al., 2020). Throughout gestation, a high expression of ACE2 in the human placenta, particularly in the decidua, the syncytiotrophoblast, and the villous stroma, may increase Ang 1–7 blood levels. Specifically, Liu D. et al. (2020) reported that ACE2 expression was upregulated between 6 and 16 weeks and downregulated in term human placentas. Plasmatic levels of ACE2 and Ang 1–7 are significantly augmented during pregnancy (Emanuele et al., 2002; Tamanna et al., 2020; Nobrega Cruz et al., 2021). On the other hand, Ang II was also increased in pregnancy (Emanuele et al., 2002). In this physiological scenario, it is believed that increased expression of ACE2 and blood levels of Ang 1–7 could be counteracting the increased stimulus of Ang II levels. Alternatively, the highest prevalence of a monomeric form of AT1, which is less sensitive to Ang II, may also reduce vascular sensitivity to Ang II. These changes would participate in the adaptive physiological mechanisms of the cardiovascular system during pregnancy, resulting in decreased peripheral vascular resistance and vasodilation of the maternal vasculature (Gant et al., 1973; AbdAlla et al., 2001; Levy et al., 2008; Marques et al., 2011; Pringle et al., 2011; Stettner et al., 2013), increased aldosterone and Ang II, promoting water and sodium retention, and increased blood volume (Scaife and Mohaupt, 2017). In addition, other critical factors modulated by the renin-angiotensin axis, increased during pregnancy, include the vasodilators prostaglandin E2, nitric oxide (Gant et al., 1980; Corthorn et al., 2006), and bradykinin (Knock and Poston, 1996). Therefore, pregnancy constitutes a physiological condition with major vascular adaptations characterized by reduced systemic vascular resistance that allows the homeostatic control of pregnancy-related hemodynamic changes, including increased cardiac output, expanded blood volume, and reduced blood pressure. Relevance of the renin-angiotensin axis in pregnancy is also remarkable in conditions in which deregulation of this axis impairs endothelial function and leads to pregnancy complications, such as hypertension or pre-eclampsia (Anton et al., 2008; Gilbert et al., 2008). Thus, it is not surprising that pregnant women constitute a potentially vulnerable population in the COVID-19 pandemic, with initial results indicating that the clinical response of pregnant women to COVID-19 could be related to physiological changes in expression levels of ACE2 and reduced sensitivity to Ang II (Anton et al., 2008; Gilbert et al., 2008; Liu D. et al., 2020; Mendoza et al., 2020).

## Clinical Presentation of Severe Acute Respiratory Syndrome Coronavirus 2 Infection in Pregnant Women

Some studies have reported that SARS-CoV-2 infection in pregnant women behaves similarly to the general population, contrary to what has been reported with other types of coronavirus infection in the past (Li N. et al., 2020; Yang et al., 2020; Fan et al., 2021). The disease manifests itself with typical symptoms and occurs with different degrees of severity, such as mild disease in 81–86% of cases, severe disease in 9.3–14%, and critical disease in 5%; values close to those reported in the general population (80, 15, and 5%, respectively) (Han et al., 2020). The most common symptoms were fever (65%), cough (60%), and shortness of breath or dyspnea (24%) (Han et al., 2020). Around 5% of mothers were admitted to the ICU; intubation was carried out in 35.87% of patients (Han et al., 2020). The rate of maternal death was <0.01%. Nevertheless, another study estimated the mortality rate in the pregnant population close to 2.7% and an ICU admission rate of 6–8% (Lambelet et al., 2020). In addition, Ellington et al. (2020) reported similar symptoms, with a greater risk of hospitalization, ICU intervention, and mechanical ventilation requirement, yet without an increased risk of death, in pregnant women when compared to non-pregnant women. It is noteworthy that, in this cohort, the group of pregnant women reported a higher frequency of comorbidities (chronic lung disease 22%, diabetes mellitus 15%, and cardiovascular disease 14%) when compared to non-pregnant women (Ellington et al., 2020).

**FIGURE 2:**
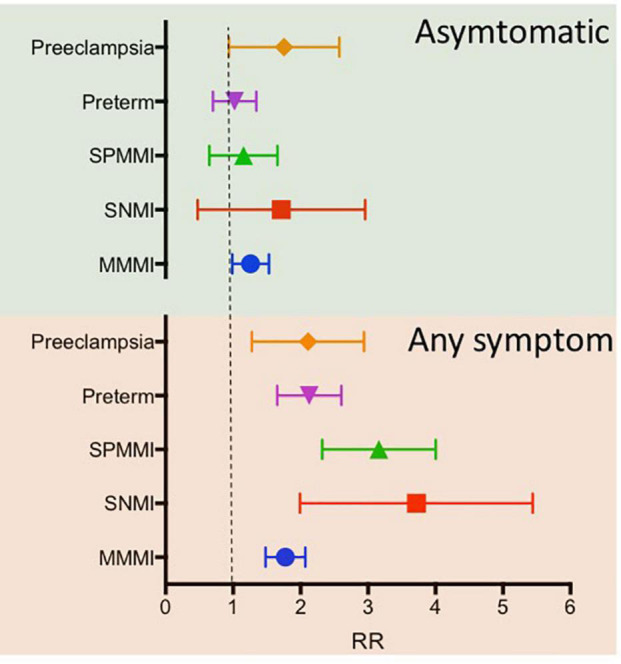
Adjusted associations for maternal and perinatal outcomes among women with coronavirus disease 2019 (COVID-19) diagnosis according to symptom status. Adjusted models for the country, month entering study, maternal age, and history of maternal morbidity (such as diabetes, thyroid, and other endocrine disorders, cardiac disease, hypertension, chronic respiratory disease, kidney disease, malaria, or tuberculosis). The upper panel includes asymptomatic COVID-19 positive patients. At the same time, the bottom panel indicates COVID-19 patients with any symptoms. Pre-eclampsia includes a diagnosis of eclampsia and/or HELLP syndrome. HELLP, hemolysis, elevated liver enzymes, low platelet count; MMMI, maternal morbidity and mortality index; RR, relative risk; SNMI, severe neonatal morbidity index; SPMMI, severe perinatal morbidity and mortality index. Data extracted from Villar et al. (2021).

Severe Acute Respiratory Syndrome and MERS have been associated with miscarriage, intrauterine death, fetal growth restriction, and high case fatality rates (Wang et al., 2021). This linkage was also presented in the pandemic of SARS-CoV-2. Thus, infection in pregnant women was associated with a high risk of adverse pregnancy outcomes, such as intrauterine growth restriction, premature rupture of membranes, fetal distress, preterm delivery (Mullins et al., 2020), spontaneous abortion, and stillbirth (Della Gatta et al., 2020). In particular, Han et al. (2020) reported that premature delivery reached 25%. The rate of low birth weight (<2,500 g) was close to 31%, and neonatal intensive care unit (NICU) admission was 24%. Positive nasopharynx swabs or sputum from newborns was <0.01% (Han et al., 2020; Figure 2). More recently, a meta-analysis published by Wei et al. (2021) included 42 studies that involve 438,548 pregnant women. They found that COVID-19 was associated with pre-eclampsia [odds ratio (OR) 1.33], preterm birth (OR 1.82), and stillbirth (OR 2.11). In addition, they found that when compared with mild COVID-19, severe COVID-19 was strongly associated with pre-eclampsia (OR 4.16), preterm birth (OR 4.29), gestational diabetes (OR 1.99), and low birth weight (OR 1.89) (Wei et al., 2021). Another meta-analysis, including twenty-eight studies with 790,954 pregnant women, concluded that SARS-CoV-2 infection during pregnancy was associated with a 58% increased risk of pre-eclampsia. In addition, there was a statistically significant increase in the risk of pre-eclampsia with severe features (OR 1.76, *p* < 0.05), eclampsia (OR 1.97, *p* < 0.05), and Hemolysis, Elevated Liver enzymes, and Low Platelets (HELLP) syndrome (OR 2.10, *p* < 0.05) among pregnant women with SARS-CoV-2 infection, as compared to those without the infection (Papageorghiou et al., 2021). In addition, there is a high prevalence of cesarean delivery, whose main indication seems to be an underlying obstetric condition, such as pre-eclampsia, fetal distress, or premature rupture of membranes, and not the clinical condition of COVID-19 in pregnant women (Della Gatta et al., 2020; Trad et al., 2020).

Conversely, other studies reported that SARS-CoV-2 infection during the first trimester of pregnancy does not seem to predispose to early pregnancy loss (Cosma et al., 2020; Rotshenker-Olshinka et al., 2020; Bortoletto et al., 2021). In addition, Rizzo et al. (2021) reported that SARS-CoV-2 infection did not increase the risk of developing fetal growth restriction. At the same time, Meyer R. et al. (2021) showed a decrease in preterm delivery in pregnant women in Israel, possibly due to the reduction of iatrogenic preterm births, avoidance of infections, or reduced stress levels related to the lockdown policy (Meyer R. et al., 2021). However, the question about the differentiation between spontaneous or iatrogenic preterm delivery remains to be elucidated (Della Gatta et al., 2020). Moreover, there was no association between abnormal umbilical artery Doppler results, defined as a composite of increased S/D ratio, absent end-diastolic velocity, reversed end-diastolic velocity, and COVID-19 infection in growth-restricted pregnancies (Ona et al., 2021).

Differences in these reports depend on several factors. First, we propose that it is relevant to consider that a significant percentage of infected pregnant women could be asymptomatic or become undiagnosed. Remarking this issue, Sutton et al. (2020) reported that in a screening testing of COVID-19 in 215 pregnant women in New York (United States), 211 women were asymptomatic, but 13.7% tested positive to SARS-CoV-2. Thus, the prevalence of COVID-19 in pregnant women may be underestimated and therefore the associated perinatal complications (Lambelet et al., 2020; Sutton et al., 2020).

## Severe Acute Respiratory Syndrome Coronavirus 2 in Latin America

**FIGURE 3:**
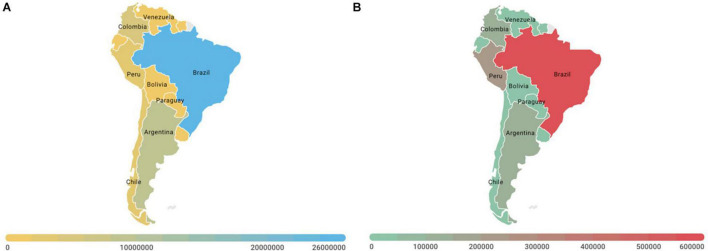
Morbi-mortality associated with coronavirus disease (COVID-19) in Latin America. **(A)** Total confirmed cases and **(B)** total mortality in Latin American countries at February 3, 2022. Brazil is the Latin American country affected the most by the COVID-19 pandemic. As of February 2022, the country had reported over 26 million cases, and 630 thousand of deaths. Source WHO; CDC; ECDC; NHC; DXY.cn; Johns Hopkins University.

There are several publications about the SARS-CoV-2 pandemic in Latin America. Most of the articles are reports of a few cases in local hospitals. However, the Iberoamerican Society of Neonatology (SABEN) recruited women with SARS-CoV-2 infection to provide knowledge and experiences on perinatal COVID-19 in Latin America (Sola et al., 2020). Their results described 86 pregnant women with COVID-19 from 11 units of 7 countries: Argentina, Colombia, Ecuador, Equatorial Guinea, Honduras, Peru, and the Dominican Republic. Of these, 68% women were asymptomatic for COVID-19, and 32% women exhibited symptoms. In total, 89% of symptomatic women had mild symptoms or signs, while 3.5% women had severe respiratory symptoms. Six women were admitted to intensive care, and no woman died. In addition, 94% were term, and 6% were preterm pregnancies. The swab result was positive in 7% of the newborns, with no causalities. Nevertheless, another study that includes 40 obstetric patients diagnosed with COVID-19 from four countries (Peru, Colombia, Bolivia, and Paraguay) reported a maternal mortality rate of 15% (six cases) and a perinatal mortality rate of 2.5% (one case). Associated pathologies included severe pre-eclampsia (25%), HELLP (5%), and gestational hypertension (12.5%), while ten patients received invasive mechanical ventilation since admission to ICU (25%) (Viruez-Soto et al., 2021; Figure 3).

In Hispanic women, obesity, advanced maternal age, medical comorbidities, and antepartum admissions related to COVID-19 have been reported as risk factors associated with adverse maternal and neonatal outcomes (Brandt et al., 2020). In Brazil, a report identified 20 COVID-19-related maternal deaths. Symptoms onset was reported during pregnancy for 12 cases, post-partum for three cases, and during the cesarean section for one case (missing data for four). In 16 cases, death occurred in the post-partum period. At least one comorbidity or risk factor was present in 11 cases (missing data for four). Asthma was the most common risk factor (5/11) (Takemoto et al., 2020).

Although Latin America has one of the highest COVID-19 death rates in the world (Editorial The Lancet, 2020), the data worldwide do not show a severe impact on maternal and fetal health (Sánchez-Duque et al., 2020). However, it is worth mentioning that only severe and critically ill patients have been tested for COVID-19, so the number of infected people (including pregnant and post-partum women) is more likely underestimated (Takemoto et al., 2020).

## Placental Infection by Severe Acute Respiratory Syndrome Coronavirus 2 and Potential Pathophysiological Mechanism

There is a significant concern regarding eventual transplacental transmission, infection at delivery or through breast milk, and the general care measures for pregnant women and neonates are being systematized. Indeed, convincing pieces of evidence have detected SARS-CoV-2 in the human placenta (Algarroba et al., 2020a; Baud et al., 2020; Best Rocha et al., 2020; Chen et al., 2020; Fenizia et al., 2020; Ferraiolo et al., 2020; Hecht et al., 2020; Hosier et al., 2020; Kirtsman et al., 2020; Kulkarni et al., 2020; Patanè et al., 2020; Richtmann et al., 2020; Smithgall et al., 2020; Vivanti et al., 2020; Hsu et al., 2021; Marinho et al., 2021). Regarding localization of SARS-CoV-2 in the placenta, most of the studies with positive results detected the presence of the virus in the syncytiotrophoblast (Best Rocha et al., 2020; Hecht et al., 2020; Hosier et al., 2020; Patanè et al., 2020; Sisman et al., 2020; Vivanti et al., 2020) and only the study of Hsu et al. (2021) detected the presence of SARS-CoV-2 in the fetal endothelium. Despite that, other studies did not detect SARS-CoV-2 in the placenta (Edlow et al., 2020; Flores-Pliego et al., 2021; Halici-Ozturk et al., 2021; Levitan et al., 2021; Table 1). Therefore, although data are not consistent, there may be vertical transmission in some cases.

**TABLE 1:** Studies that assess the detection of Severe Acute Respiratory Syndrome Coronavirus 2 (SARS-CoV-2) in placenta around the world indicate the technique used and the state of symptoms.

Method of detection of SARS-CoV-2 in placenta				Presence of COVID-19 symptoms		Total cases	Country	References
	
qPCR	IHC	ISH	EM	+	−			
Neg	ND	ND	ND	1		1	Turkey	Kalafat et al., 2020
Neg	ND	ND	ND	1		1	China	Peng et al., 2020
Pos(1)	ND	ND	ND	11		11	United States	Penfield et al., 2020
ND	ND	ND	Pos	1		1	United States	Algarroba et al., 2020b
ND	Pos(1)	Pos(2)	ND	ND	ND	19	United States	Hecht et al., 2020
ND	Pos	ND	ND		1	1	United States	Hsu et al., 2021
Pos	ND	ND	ND	1		1	India	Kulkarni et al., 2020
Pos	ND	ND	ND	4	1	5	Brazil	Richtmann et al., 2020
ND	Neg	Neg	ND	50	26	76	United States	Smithgall et al., 2020
Pos	Pos	ND	ND	ND	ND	1	United States	Vivanti et al., 2020
ND	Pos	ND	Pos	1		1	United States	Sisman et al., 2020
ND	Pos	Pos	ND	1	1	2	United States	Best Rocha et al., 2020
Pos	ND	ND	ND		1	1	Italy	Ferraiolo et al., 2020
Pos	Pos	Pos	Pos	1		1	United States	Hosier et al., 2020
ND	Pos	Pos(2)	ND	3		3	Italy	Patanè et al., 2020
Pos	ND	ND	ND	3		3	China	Chen et al., 2020
Pos	ND	ND	ND	1		1	Switzerland	Baud et al., 2020
Pos	ND	ND	ND	1		1	Canada	Kirtsman et al., 2020
Pos (2)	ND	ND	ND	31		31	Italy	Fenizia et al., 2020
ND	ND	Neg	ND	15	32	47	United States	Edlow et al., 2020
Neg	ND	ND	ND	11		11	Mexico	Flores-Pliego et al., 2021
Neg	ND	ND	ND	5	19	24	Turkey	Halici-Ozturk et al., 2021
Pos	Pos	Pos	Pos	1		1	Mexico	Valdespino-Vázquez et al., 2021

*qPCR, real-time PCR; IHC, immunohistochemistry; ISH, In situ hybridization; EM, electron microscopy; pos, positive; Neg: negative; ND, not determined.*

Interestingly, there is also evidence for *in utero* transmission of SARS-CoV-2 infection. For instance, detection of SARS-CoV-2 virus in nasopharyngeal samples of newborns and anti-SARS-CoV-2 antibodies detected in the umbilical cord blood in at least one case were reported (Fenizia et al., 2020). In addition, Sisman et al. (2020) presented a preterm infant with placental SARS-CoV-2 infection and positive nasopharyngeal testing at 24 and 48 h of life, who developed fever and mild respiratory disease on the second day of life.

Whether ACE2 and TMPRSS2 are entry mechanisms for SARS-CoV-2 in the placenta remains unclear, although both proteins are expressed in the trophoblast and fetal endothelium. Thus, ACE2 was detected in syncytiotrophoblast (Edlow et al., 2020; Hecht et al., 2020; Taglauer et al., 2020; Lu-Culligan et al., 2021) and endothelium of umbilical arteries (Valdés et al., 2006). Nevertheless, a study conducted in isolated cells from the placenta shows that the co-expression of ACE2 and TMPRSS2 is very low in syncytiotrophoblast, cytotrophoblast, and extravillous trophoblast, especially when it is compared with the expression of cytomegalovirus and Zika virus receptors. These pathogens have been proven to cross the placenta and generate vertical transmission to the fetus (Pique-Regi et al., 2020). In addition, Li M. et al. (2020) reported high expression of ACE2 and TMPRSS2 in syncytiotrophoblast and cytotrophoblast in the single-cell evaluation of trophoblasts in the human placenta. Despite this evidence, it is necessary to investigate whether the expression of both proteins in the placenta may be affected in pregnancy complicated by SARS-CoV-2 infection since a weak expression of TMPRSS2 in the villous endothelium of SARS-CoV-2 positive pregnant women was reported (Edlow et al., 2020; Hecht et al., 2020).

Additionally, fetal sex could affect the placental expression of ACE2. It has been reported that the male sex is associated with a higher ACE2 gene expression (Chlamydas et al., 2021). Moreover, it is known that the ACE2 gene escapes X inactivation (Tukiainen et al., 2017), *in vitro* study shows that 17β-estradiol, a primarily female sex steroid, can downregulate the ACE2 gene expression in non-pregnant women (Stelzig et al., 2020), providing another hypothesis for the sex-based expression differences of this gene. However, there is no information about sex-dimorphism in the placental expression of ACE2 in pregnant women with COVID-19.

On the other hand, detecting SARS-CoV-2 in placentas from fetal death cases alerted the possible occurrence of placental dysfunction associated with the maternal medical condition (Baud et al., 2020; Hosier et al., 2020; Richtmann et al., 2020). These studies suggest that early infection (first or second trimester of pregnancy) could generate a state of more significant deterioration in the placental function associated with exacerbated inflammation, negatively impacting fetal wellbeing and development. Supporting this pathophysiological explanation, a recent case report from Brazil showed an association of fetal death at 34 weeks of gestation with histopathological alterations in the placenta. The findings are consistent with intense acute placenta vascular malperfusion, with the detection of SARS-CoV-2 in the placenta, umbilical cord, and fetal tissues (lung, heart, and brain, among others). Furthermore, the patient had mild symptoms of COVID-19 but, at the time of fetal death, had pro-inflammatory and pro-coagulant syndrome characterized by higher levels of interleukins, ferritin, and D-dimer (Marinho et al., 2021). Therefore, further research is needed to elucidate the involvement of placental dysfunction in COVID-19 cases and its perinatal consequences.

## Placental Vascular Disorders Associated With Severe Acute Respiratory Syndrome Coronavirus 2

Regarding the vascular alterations of the placenta, histological studies of placentas from SARS-CoV-2 positive pregnant women showed poor uterine-placental perfusion, with signs of placental infarcts, atheromas in decidua vessels, chorioangioma, and edema in the placental villi (Mulvey et al., 2020; Shanes et al., 2020). Those studies also associated vascular thrombosis in the chorionic plate and decreased capillaries density in the chorionic villi in placentas with SARS-CoV-2 infection. Moreover, Baergen and Heller (2020) described that this viral infection was associated with a high risk of fetal vessel thrombosis and reduced vascularization in the placental microcirculation. Similarly, Patberg et al. (2020) showed that SARS-CoV-2 infection is associated with placental signs of reduced perfusion and villitis of unknown etiology. Another study confirmed these findings, including asymptomatic or mildly symptomatic SARS-CoV-2 positive pregnant women, showing evidence of fetal vascular malperfusion (FVM): chorioangiosis, intramural fibrin deposition, and vascular ectasia. Additionally, perivillous fibrin deposition was also significantly higher in placental histopathology (Jaiswal et al., 2021). These results agree with Meyer J. et al.’s (2021) findings showing that 77% of placentas infected with SARS-CoV-2 showed one or more features of maternal vascular malperfusion (MVM). Therefore, growing pieces of evidence, with some exceptions (Zhang et al., 2020; Levitan et al., 2021; Lu-Culligan et al., 2021), have shown placental vascular disorders in pregnant women with COVID-19.

**FIGURE 4:**
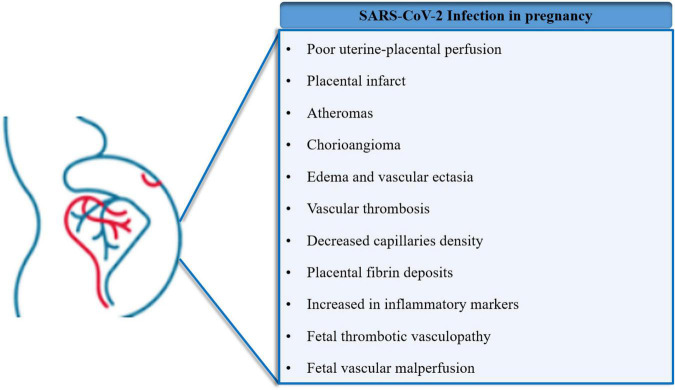
Major placental and gestational disorders associated with Severe Acute Respiratory Syndrome Coronavirus 2 (SARS-CoV-2). Pregnant women constitute a population group with special conditions due to the imminent risks to the mother and fetus. Recent evidence has shown that SARS-CoV-2 infection during pregnancy results in distinct physiological alterations, affecting placental function and distinct gestational parameters.

Whether the severity of COVID-19 in pregnancy is related to placental alterations is under investigation. For instance, high trophoblast necrosis was found in pregnant women who required respiratory support or intubation for COVID-19 when compared with non-hypoxic patients (Meyer J. et al., 2021). Moreover, Edlow et al. (2020) found that placental lesions associated with MVM were increased with the severity of COVID-19 in pregnant women. Remarkably, in patients with COVID-19 admitted to the ICU, increased levels of von Willebrand Factor (vWF) antigen and P-selectin in plasma were detected, indicating endothelial dysfunction (Goshua et al., 2020). In addition, in the placenta of pregnant women with severe COVID-19, higher expression of vWF was associated with lower claudin-5 and vascular endothelial (VE-cadherin) in the endothelium from decidua and chorionic villi (Flores-Pliego et al., 2021). These findings suggest that COVID-19 induces endothelial cell injury in the placenta, probably affecting the endothelial barrier, the anti-thrombotic capacity of the endothelium, and the overall function of placental vessels. Compromised placental function, placental hypoxia, and a hypercoagulable state are probably related to the severity of the infection (Ng et al., 2006; Baergen and Heller, 2020; Shanes et al., 2020; Marinho et al., 2021; Meyer J. et al., 2021; Poisson and Pierone, 2021; Figure 4).

In addition, SARS-CoV-2 positive mothers showed higher levels of inflammatory markers. Thus, Fenizia et al. (2020) showed significant increases in inflammatory markers (including interleukin 1β or interleukin 6) in both placenta and plasma of two women whose placenta was tested positive for SARS-CoV-2. Accordingly, it was previously reported that the presence of SARS-CoV-2 in the placenta generated an increased inflammatory response in the intervillous space (histiocytic intervillositis) and chorionic villi (villositis), with the presence of macrophages (CD68) and T lymphocytes (CD3) in the intervillous space (Hecht et al., 2020; Hosier et al., 2020; Kirtsman et al., 2020; Patanè et al., 2020; Sisman et al., 2020; Smithgall et al., 2020; Vivanti et al., 2020; Hsu et al., 2021). Transcriptome analysis of placentas from pregnant women with COVID-19 showed increased genes associated with immune response. Specifically, Lu-Culligan et al. (2021) found a marked increase of pro-inflammatory genes and chemokines in both immune and non-immune cell types in placentas from COVID-19 cases. In addition, single-cell transcriptome analysis revealed significant enrichment of genes encoding cytotoxic proteins in natural killer (NK) cells, associated with upregulation of the activation marker CD69 in T-cells, increased expression of interferon-induced protein ISG15, and the regulators of nuclear factor-κB (NFκB) pathway in endothelial cells (Lu-Culligan et al., 2021).

Despite these findings, the potential implications of vascular alterations of the placenta in COVID-19 cases and fetal development are not entirely understood (Ahlberg et al., 2020; Díaz-Corvillón et al., 2020; Khalil et al., 2020; Shanes et al., 2020). Therefore, there is a demanding necessity to continue gathering information, aiming to elucidate the potential implication of impaired placental environment observed in women with COVID-19 in contributing to adverse perinatal outcomes, particularly in periods of the high spread of the pandemic.

## The Impact of Coronavirus Disease 2019 Vaccine on Pregnancy

Pregnant women have traditionally been excluded from vaccine trials. Without appropriate evidence about safety and efficacy during pregnancy, they have previously been denied the opportunity to receive vaccines that would have protected them and their offspring. This situation has also been present in the context of the COVID-19 pandemic, and pregnant women, their providers, and health policymakers would have to make unnecessarily tricky decisions because of inadequate evidence about vaccine use in pregnancy. This would lead to less vaccine use and its afforded protections in this population (Beigi et al., 2021). In the V-SAVE pregnancy registry with 3,958 participants who received messenger RNA (mRNA) COVID-19 vaccines, 827 had a completed pregnancy, of which 115 (13.9%) resulted in a pregnancy loss and 712 (86.1%) resulted in a live birth (mostly among pregnant women vaccinated in the third trimester). Adverse neonatal outcomes included preterm birth (in 9.4%) and small size for gestational age (in 3.2%), and no neonatal deaths were observed. Although not directly comparable, calculated proportions of adverse pregnancy and neonatal outcomes in persons vaccinated against COVID-19 who had a completed pregnancy were similar to incidences reported in studies involving pregnant women that were conducted before the pandemic (Shimabukuro et al., 2021). On the other hand, a recent study in 24,288 singleton pregnancies showed no evident differences, in terms of adverse neonatal and early infant outcomes, between newborns of women who received BNT162b2 mRNA vaccination during pregnancy vs. those of women who were not vaccinated. This study contributes to current evidence in establishing the safety of prenatal vaccine exposure to newborns. However, the interpretation of study findings is limited by the observational design (Goldshtein et al., 2022).

Clinical trials demonstrate that vaccination effectively prevents severity and symptomatic COVID-19 in non-pregnant persons. To highlight, the risks of administering COVID-19 vaccines during pregnancy appear to be minimal. Commonly reported side effects are short-term injection site pain, headache, fever, myalgia, arthralgia, chills, and nausea (National Center for Immunization and Respiratory Diseases, 2021). Moreover, the rate of serious adverse effects has been relatively low (National Center for Immunization and Respiratory Diseases, 2021). In addition, chemical components of the vaccines are not specifically contraindicated in pregnancy (National Center for Immunization and Respiratory Diseases, 2021). About the recommendations, the American College of Obstetrics and Gynecology states that in the absence of data showing that vaccines are contraindicated, then pregnant patients should be immunized (American College of Obstetricians and Gynecologists’ Immunization Infectious Disease and Public Health Preparedness Expert Work Group et al., 2021). The Center for Disease Control has taken a similar position, declaring that the only absolute contraindication to vaccination is an allergy to vaccine components (National Center for Immunization and Respiratory Diseases, 2021). However, the World Health Organization has more reserved recommendations, indicating that vaccination is only indicated in pregnant women who are at high risk for exposure to COVID-19 (healthcare workers, or those with comorbidities that might make disease more severe) (Chavan et al., 2021; World Health Organization, 2021).

## Concluding Remarks

In this manuscript, we have discussed the vulnerability of the pregnant population to COVID-19 infection. This manuscript also described many uncertainties; however, they are not the only ones in this field. For example, the real incidence of COVID-19 in pregnant women is a fundamental question that needs clarification. In addition, whether COVID-19 is associated with a greater risk of severe disease and perinatal complications requires confirmatory population studies. This information would help to generate appropriate public health policies for this particular population.

In addition, we also have remarked that infected placenta with SARS-CoV-2 showed alterations related to inflammatory processes associated with damages to the vascular network. Whether these placental alterations might explain the adverse perinatal outcomes in women with COVID-19 requires confirmatory studies. In addition, although there is vertical transmission in some cases, vertical transmission data are not consistent. We encourage future research to elucidate whether SARS-CoV-2 infection affects fetal programming, as well as the future health of both mother and offspring.

Concerning the vaccine, pregnant women and physicians need to use the limited available data to weigh the benefits and risks of the COVID-19 vaccine during pregnancy, considering the patient’s specific risk of SARS-CoV-2 exposure. Currently, there is the absence of evidence that supports pregnancy as a contraindication, and it seems that the benefits of receiving the vaccine far outweigh the unlikely potential harms. However, estimates of global vaccination among pregnant women are yet unknown. Moreover, we remark inequities in the access to the SARS-CoV-2 vaccines worldwide, but, in particular, in Latin America. For example, only 56% of the Latin American people have been vaccinated up to December 2021 (PAHO, 2021), with a significant difference among countries (PAHO, 2022). In addition, it is necessary to delve into how the pandemic has impacted the care of pregnant women in general since substantial and heterogeneous modifications have been reported in maternity services (Jardine et al., 2021).

In conclusion, the available information highlights the greater vulnerability of pregnant women in the context of a pandemic. However, more studies are required to better understand the potential impact of the pandemic on pregnant women, especially in Latin American nations.

## Author Contributions

PA-R, MG, and RG-R conceived, designed, planned, and supervised the manuscript. PA-R, MG, and CE critically review the manuscript and generate the final published version. LQ-A, FG, RA, CE, and AD wrote the manuscript. All authors provided critical feedback and approved the final version.

## Conflict of Interest

The authors declare that the research was conducted in the absence of any commercial or financial relationships that could be construed as a potential conflict of interest.

## Publisher’s Note

All claims expressed in this article are solely those of the authors and do not necessarily represent those of their affiliated organizations, or those of the publisher, the editors and the reviewers. Any product that may be evaluated in this article, or claim that may be made by its manufacturer, is not guaranteed or endorsed by the publisher.
